# Concomitant predation on parasites is highly variable but constrains the ways in which parasites contribute to food web structure

**DOI:** 10.1111/1365-2656.12323

**Published:** 2015-01-08

**Authors:** Alyssa R. Cirtwill, Daniel B. Stouffer

**Affiliations:** ^1^ School of Biological SciencesUniversity of CanterburyChristchurchNew Zealand

**Keywords:** interaction roles, network motifs, role dispersion, role diversity, species roles

## Abstract

1. Previous analyses of empirical food webs (the networks of who eats whom in a community) have revealed that parasites exert a strong influence over observed food web structure and alter many network properties such as connectance and degree distributions. It remains unclear, however, whether these community‐level effects are fully explained by differences in the ways that parasites and free‐living species interact within a food web.
2. To rigorously quantify the interrelationship between food web structure, the types of species in a web and the distinct types of feeding links between them, we introduce a shared methodology to quantify the structural roles of both species and feeding links. Roles are quantified based on the frequencies with which a species (or link) appears in different food web motifs – the building blocks of networks.
3. We hypothesized that different types of species (e.g. top predators, basal resources, parasites) and different types of links between species (e.g. classic predation, parasitism, concomitant predation on parasites along with their hosts) will show characteristic differences in their food web roles.
4. We found that parasites do indeed have unique structural roles in food webs. Moreover, we demonstrate that different types of feeding links (e.g. parasitism, predation or concomitant predation) are distributed differently in a food web context. More than any other interaction type, concomitant predation appears to constrain the roles of parasites. In contrast, concomitant predation links themselves have more variable roles than any other type of interaction.
5. Together, our results provide a novel perspective on how both species and feeding link composition shape the structure of an ecological community and vice versa.

## Introduction

Food webs – the networks of who eats whom in an ecosystem – provide ecologists with tools to analyse the structure of ecological communities (Cohen 1978; Pascual & Dunne 2006) and compare them across space and time (Thompson & Townsend 2005b; Shurin, Gruner & Hillebrand 2006; Olesen *et al*. 2008). Food webs also connect biodiversity to ecosystem functions by integrating patterns and processes from individual to community scales (Thompson *et al*. 2012). In particular, the overall structure of food webs has been directly tied to ecosystems' responses to environmental change (Thompson & Townsend 2003, 2005a; Tylianakis *et al*. 2008) and robustness to species loss (Dunne, Williams & Martinez 2002a, 2004; Estrada 2007; Srinivasan *et al*. 2007; Gilbert 2009; Rezende *et al*. 2009).

The vast majority of food web studies, however, have focused on networks of predator–prey interactions between free‐living species (Combes 1996; Huxham, Beaney & Raffaelli 1996; Marcogliese & Cone 1997; Lafferty, Dobson & Kuris 2006), prompting calls for a broader and more comprehensive food web theory (Marcogliese & Cone 1997; Lafferty, Dobson & Kuris 2006; Fontaine *et al*. 2011; Kéfi *et al*. 2012), especially where parasites are concerned (Marcogliese & Cone 1997; Lafferty, Dobson & Kuris 2006; Dobson *et al*. 2008; Lafferty *et al*. 2008). Although typically small and difficult to observe, parasites can exert a strong influence on their communities (e.g. Huxham, Beaney & Raffaelli 1996). They participate in a large proportion of feeding links (henceforth ‘links’) (Lafferty, Dobson & Kuris 2006; Dunne *et al*. 2013a) and exhibit comparable diversity and biomass to free‐living species (Dobson *et al*. 2008; Kuris *et al*. 2008). Moreover, parasites' complex life histories, which commonly involve different sets of hosts for different life stages, render them vulnerable to secondary extinctions and therefore decrease network robustness (Lafferty & Kuris 2009).

**Figure 1:**
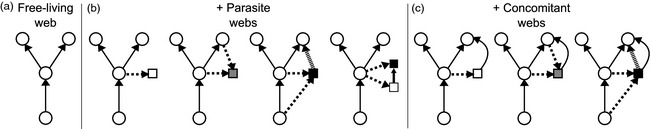
Parasites can be incorporated into food webs in several different ways, each of which increases the complexity of the web. (a) Food webs are typically composed of free‐living species (circles) and the predator–prey links between them (arrows indicate the direction of energy flow). (b) In ‘+ parasite’ webs, parasites (squares) parasitize free‐living hosts (dotted line). They may parasitize one host for their entire life cycle (white square), different hosts (grey square), or be target prey to free‐living predators (black square, hatched line). Where two parasites infect the same host (black and white square), one may kill the other, usually consuming it (thick black line). (c) ‘+ concomitant’ webs also include links between parasites and the predators of their hosts (curved lines). In these links, the parasite may simply be digested (white square), or it may infect the predator and parasitize it as well (grey square). In some cases, a parasite (black square) may be consumed by the same predator both as concomitant prey and as target prey.

Parasites are also of interest because of the many ways in which they could potentially influence food web structure – the organization of links between species (Combes 1996; Thompson, Mouritsen & Poulin 2004; Lafferty, Dobson & Kuris 2006; Dunne *et al*. 2013a; Thieltges *et al*. 2013; Fig.  1). Like generalist predators, many parasites have multiple potential hosts which may each support different life stages (Marcogliese & Cone 1997; Lafferty, Dobson & Kuris 2006; Rudolf & Lafferty 2011). Parasites may also have one or more free‐living stages which can be important prey for free‐living predators (Combes 1996; Kuris *et al*. 2008). Further, parasites vary in the ways in which they are transmitted between hosts: they can actively infect new hosts, be ingested as eggs or cysts, or be ingested as concomitant prey along with the current host (Kuris *et al*. 2008; Thieltges *et al*. 2013).

Because of their plethora of life‐history strategies, small body sizes and unusual mode of life, it would appear that the ecological roles of parasites are completely distinct from those of more ‘traditional’ predators and prey (Marcogliese & Cone 1997; Rudolf & Lafferty 2011). Indeed, at least one study has concluded that parasites tend to have broader and less‐contiguous prey ranges than free‐living species (Dunne *et al*. 2013a). Despite these important differences, however, that same study suggested that parasites and free‐living species can appear to have similar effects on food web structure. For example, when parasites are added to a food web without including concomitant predation, species richness and number of links necessarily increase and connectance, link density and degree distributions are thereby altered (Dunne *et al*. 2013a). Nevertheless, these structural changes are similar to the trends that emerge when comparing webs with different numbers of free‐living species (Dunne *et al*. 2013a) and follow known patterns of scaling with species richness (Riede *et al*. 2010).

In contrast, the addition of concomitant predation links resulted in greater structural changes. First, by adding more links but no additional species, link density and connectance must necessarily increase (Dunne *et al*. 2013a). Importantly, this increase in connectance did not fit the scaling pattern observed in free‐living webs and was observed when connectance was adjusted to account for the exclusion of concomitant predation (Dunne *et al*. 2013a). The higher connectance of food webs including concomitant links may in turn drive other trends in food web structure, especially in properties such as mean food chain length which have been observed to increase when parasites are added to food webs (Lafferty, Dobson & Kuris 2006) and are known to positively correlate with connectance (Dunne, Williams & Martinez 2002b). In addition to changing connectance, the addition of concomitant predation altered the frequencies with which different configurations of interactions among species occurred. In particular, the overlay of host–parasite and predator–prey interactions changed the frequencies of two‐way feeding interactions (A eats B and B eats A), reflecting an effect of the intimacy between host and parasite on network structure (Dunne *et al*. 2013a).

This increase in connectance and the trickle‐down effects on food web structure attributable to higher connectance suggest that parasites may have their most unique effects on food web structure as concomitant prey (Dunne *et al*. 2013a). This notion was most strongly supported by an analysis of three‐species food web motifs from the same study. A food web motif represents a unique interaction pattern such as three‐species food chains, apparent competition, or trophic loops (Milo *et al*. 2002; Kashtan *et al*. 2004; Stouffer *et al*. 2007, 2012), and the frequencies with which different motifs occur can be used to characterize fine‐scale food web structure (Stouffer *et al*. 2007). These frequencies were similar for webs composed solely of free‐living species and webs including parasites but not concomitant links (Dunne *et al*. 2013a). This implies that the roles of free‐living species serving as hosts are structurally similar to those of free‐living species serving as prey and that parasites as consumers have similar roles to free‐living consumers (Dunne *et al*. 2013a). When concomitant links were added, the frequencies of motifs including at least one two‐way link changed. This appeared to be driven by the increase in intraguild predation (predation between two species that share a common prey/host) as parasites are eaten along with their host (Dunne *et al*. 2013a), suggesting that parasites have different structural effects as resources than free‐living species.

Comparisons of whole‐network structure such as these, however, can mask the mechanisms behind the trends they uncover (Stouffer 2010) since knowledge of a network‐level pattern does not unambiguously determine how different species contribute to that pattern (Saavedra *et al*. 2011; Stouffer *et al*. 2012). For example, network‐level measures such as connectance are a useful first step to predict overall community stability (Dunne, Williams & Martinez 2002a), but connectance alone is a poor predictor of variation in species' degrees (Dunne, Williams & Martinez 2002b) or which species is most critical to maintain that stability (Dunne, Williams & Martinez 2002a; Olesen *et al*. 2011). One way to overcome this drawback is to examine network structure directly from the perspective of the building blocks of networks: species and the links between them (Stouffer 2010; Baker *et al*. 2014).

Here, we use an extension of food web motifs to quantify species' ‘structural roles’ – which provide holistic summaries of how they interconnect with the rest of the web (Stouffer *et al*. 2012; Fig. S1, Supporting information) – and hence to compare the different ways in which parasites and free‐living species are thus embedded in their communities. This definition of role is rigorously defined by the relative frequencies with which species appear across different motifs like apparent competition, omnivory, or trophic loops. As such, our definition of roles incorporates information on a species' predators and prey, as well as how that species is indirectly linked to more distant species. Roles can therefore also be conceptualized as summaries of the ‘shape’ of species' biotic niches within a food web. As a consequence, we can estimate the degree to which species' contributions to network structure (and hence to energy flows and other ecosystem functions) are redundant by identifying species with similar roles. Such species can likely compensate for each other in the face of disturbances, increasing the network's robustness (Naeem 1998; Rosenfeld 2002).

To understand how roles can vary between species, consider three hypothetical top predators: one which is a strict specialist that acts as the top of only one food chain; a second, generalist predator that acts as the top of several food chains; and a third predator that forms the top of several food chains *and* engages in omnivory. The roles of the first two predators are very similar – despite having different numbers of prey species, both predators only ever appear in one position in the food web: at the top of a food chain. The third predator, which is involved in motifs describing omnivory, as well as three‐species food chains, has a more complex role. One could therefore argue that the first two species make similar structural contributions to the network while the third predator has a distinct effect. Moreover, these species likely make different contributions to the stability and functioning of the community (Stouffer 2010; Stouffer *et al*. 2012).

This argument rests upon the fact that species' structural roles describe the ways a species directly and indirectly influences biomass and energy flows through a food web. Therefore, the hypothesis that parasites and free‐living species interact with other species in fundamentally different ways can be directly tested by comparing their structural roles. Here, we focus on the comparison of the roles of parasites to those of free‐living species interacting only with other free‐living species. When concomitant predation is excluded, parasites have many prey but few consumers and are usually considered to be the tops of their food chains (Thompson, Mouritsen & Poulin 2004). We therefore expect the structural roles of parasites excluding concomitant predation to be similar to the roles of free‐living species with no free‐living predators (hereafter ‘top predators’) or to intermediate consumers with few free‐living predators. When concomitant predation is taken into account, however, parasites have both prey and many consumers. If parasites have similarly shaped niches to those of free‐living species, we would then expect the structural roles of parasites including concomitant predation to be similar to those of free‐living intermediate consumers.

In a similar way, we can examine food webs from the perspective of the links within them. Just as a species' structural role summarizes the ways in which it is connected to other species, a link's structural role summarizes the ways in which an energy transfer between two species is embedded in the larger food web (Fig. S3, Supporting information). The roles of links, like those of species, vary depending on how many connections a link has to the rest of the web and the nature of species involved in those connections. A link between an unpalatable basal resource and a specialist herbivore which in turn interacts with a single consumer, for example, would have a role summarized by a single dimension describing its single position. In contrast, a link between two generalist intermediate consumers would have a role with many dimensions corresponding to the many disparate positions that link appears in across food web motifs. Note that, as with species, link roles describe the relative frequencies with which a link occupies different positions rather than the raw count. Thus, a link which appeared in the same position 10 times would have the same role as a link which only appeared in that position once, and both would have very different roles to a link which appeared once in each of 10 different positions. By comparing link roles in this way, we can determine whether feeding links involving parasites are organized differently within a food web regardless of whether the roles of parasites themselves are different. This alternative view is hinted at by the observation that food web structure is altered more by the inclusion of concomitant links than by the simpler addition of parasites without concomitant predation (Dunne *et al*. 2013a).

It is more difficult to generate intuitive hypotheses about differences between the roles of types of links because of a dearth of previous studies that have directly characterized their roles in food webs. Nevertheless, predation, parasitism, and concomitant predation all involve different types of species and have different functional consequences for the two interacting species. We therefore expect significant differences in the structural roles of these links. Since adding concomitant predation links changed the motif structures of food webs (Dunne *et al*. 2013a), we expect that these links will have different roles from those of links between free‐living species. Conversely, because adding links describing parasitism and predation among parasites to food webs does not change motif structure of food webs, we expect that these links will have similar roles to those of links between free‐living species.

**Figure 2:**
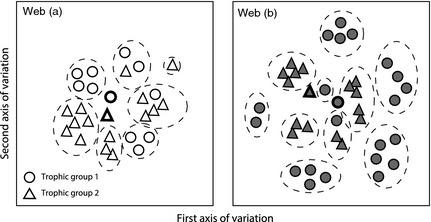
Visualizing the distribution of species roles within two hypothetical food webs (a) and (b). In the two panels, we depict the roles of two trophic groups (e.g. top predators and intermediate consumers) and indicate them by circles and triangles, respectively. Because our definition of roles is multidimensional, they are most easily represented using a correspondence analysis in which roles are compared along major axes of variation rather than axes based on particular motifs. Axis one might represent, for example, the tendency for roles to contain motifs involving two‐way interactions, while axis two might represent the tendency for roles to contain motifs representing trophic loops. Under this representation, dispersion and diversity provide complimentary measures of the distribution of roles within communities. Dispersion measures the spread of roles about the median role for a trophic group (indicated by shapes with thick outlines) while diversity measures the number of statistically identifiable role ‘phenotypes’ (indicated by dashed ovals). In hypothetical web (a), the roles of the two types of species have similar levels of dispersion and diversity despite greater numbers of species in trophic group 2 being present in the community. In hypothetical web (b), the roles of species in trophic group 1 are more widely dispersed and more diverse than those of trophic group 2.

As well as comparing roles of different types of species and links across communities, we aimed to study the variability of different roles within communities. Measuring this variability provides a more rigorous analysis of the potential overlap or redundancy among the structural roles of species within a type. Specifically, we quantified the within‐community dispersion and diversity of roles for each group of species and links. The dispersion of a type of roles is its within‐group variance – that is, how similar the roles of each group of species or links are to the median role for that group in its community (see Materials and methods). A high role dispersion for a group of species indicates that each species' role has limited overlap with those of other species in the same group. Role diversity, in contrast, quantifies the observed number of statistically unique role ‘phenotypes’ – characteristic multidimensional shapes into which roles can be grouped – occupied by species or links from a particular group in a community (see Materials and methods). Role diversity therefore offers a perspective on how different types of species or links contribute to the overall role diversity of a food web. A high diversity of roles for a group of species means that these species occupy a wider range of the potential roles available to all species in all food webs. Importantly, these two measures are complimentary, such that a group of species whose roles have high dispersion might exhibit high or low role diversity (Fig. 2).

Once the distributions of species and link roles have been quantified within communities, we are able to compare these distributions across communities. Similar patterns of distribution across communities can point to general rules in food web structure such as the scaling of many food web properties with species richness and connectance (Havens 1992; Dunne, Williams & Martinez 2002b; Riede *et al*. 2010). Here, we are particularly interested in whether role dispersion and diversity exhibit scaling relationships with species richness (or link richness, in the case of link roles). If, for example, dispersion and diversity increase with species richness, this would suggest that species roles are increasingly variable in larger webs and that adding more species does not create redundancy within the food web. Such a situation would recall May's ‘devious strategies’ by which communities persist, with none acting in the exact same way as the next (May 2001). It is also possible that role dispersion and diversity do not increase with species or link richness; such saturation of role distributions would indicate high redundancy and create a community that is robust to perturbations (Petchey *et al*. 2008).

## Materials and methods

### Empirical data

The food webs studied here describe seven temperate coastal communities (Huxham, Beaney & Raffaelli 1996; Hechinger *et al*. 2011b; Mouritsen *et al*. 2011; Thieltges *et al*. 2011; Zander *et al*. 2011; Tables S1–S3) that included both free‐living species and parasites (see Appendix S1 for the full definition of ‘parasite’). Since we were interested in particular species rather than whole‐network characteristics, we did not aggregate species with the same predator and prey sets into trophic species as is common elsewhere (Martinez 1991; Vermaat, Dunne & Gilbert 2009; Dunne *et al*. 2013a). The links in these food webs describe several different classes of interaction: predation among free‐living species, parasitism of free‐living species, predation among parasites, and target and concomitant consumption of parasites (Hechinger *et al*. 2011b).

Using these different link types, we constructed three food webs describing different interactions among the species in each community (Fig. 1). The first, ‘free‐living’ web contains only free‐living species and the predator–prey links between them. The second, ‘+ parasite’ web includes every species and link in the free‐living web as well as parasites, parasitism of free‐living species, intraguild predation between parasites, and predation by free‐living species upon parasites in which the parasite is target prey (e.g. when a fish consumes trematode cercariae). The third and most complex, ‘+ concomitant’ web contained all of the species and links in both of the previous webs as well as concomitant links where parasites are consumed together with their hosts. For each of the seven communities, we therefore have a free‐living, parasite, and concomitant web (giving a total of 21 food webs).

### Quantifying species roles

We then analysed the role of each species within its community by quantifying the ways in which the focal species participates in the set of 13 network motifs – unique three‐species building blocks that make up a food web (Milo *et al*. 2002; Kashtan *et al*. 2004; Stouffer *et al*. 2007, 2012). Of the three‐species motifs, five contain only one‐way interactions (A eats B, B does not eat A) and the remaining eight contain at least one two‐way interaction (A eats B and B eats A). The two types of motifs tend to occur with different frequencies (Stouffer *et al*. 2007) and, by definition, have different effects on energy flow throughout a food web. The frequency with which a species appears in each motif summarizes the organization of its feeding links, as both predator and prey. Mathematically, the number of times a focal species *i* in community *s* (e.g. the Ythan estuary) in web type *w* (e.g. the ‘+ parasite’ web) appears in each of the 30 unique positions across the 13 three‐species motifs gives a multidimensional vector $\vec{f_{si}^{w}}$ that robustly quantifies the species' role within the food web (Stouffer *et al*. 2012; Appendix S2, Fig. S1, Supporting information).

Having calculated the set of roles for all species in all webs for each community, we first compared the roles for species in different trophic groups. We divided free‐living species into top predators (T), basal resources (B) and intermediate consumers (I) based on their interactions with other free‐living species (see Appendix S1 for more details). Since food webs have traditionally been composed only of free‐living species and the roles of species have been understood in this context, we used the roles of free‐living species in the free‐living webs as a baseline against which to compare the roles of parasites with (P${}_{c}$) and without (P) concomitant links. Although using the free‐living species web as a baseline means comparing the roles of parasites in a larger web to free‐living species in a smaller web, network‐level results suggest that motif frequencies do not change systematically after the addition of more species, including parasites (Bascompte & Melián 2005; Stouffer *et al*. 2007; Dunne *et al*. 2013a). We therefore do not expect network size to greatly influence parasites' roles compared to those of free‐living species. We included the roles of the same parasite species in both the ‘+ parasite’ and ‘+ concomitant’ webs in order to determine whether parasites have different roles when concomitant links are excluded or included. All five groups of species were represented in each of the seven webs, giving a sample size of *n* = 35 for analysis of species roles.

### Quantifying link roles

Following an analogous methodology to that used in the determination of species roles, each link *k* in web type *w* at community *s* was assigned a role vector $\vec{f_{sk}^{w}}$ based on the frequency with which it occurred in each of the 24 unique ‘link positions’ that make up the 13 three‐species motifs (Appendix S2, Fig. S2). As with the roles of species, we used links between free‐living species (F→F) in the free‐living webs to set the *de facto* baseline since these are the links current food web theory is based upon. For consistency with the analysis of species roles, we included the roles of all other types of links from the least complex web in which they appeared. That is, we used the roles of parasitism (F→P), intraguild predation (P→P) and target predation on parasites (P$\overset{t}{\to }$F) as calculated in the ‘+ parasite’ webs and the roles of concomitant predation (P$\overset{c}{\to }$F) links from the ‘+ concomitant’ webs. P$\overset{c}{\to }$F links include those in which the ingested parasite can infect its predator (i.e. trophic transmission) and those in which the parasite is digested and killed. Note that predation among parasites and target predation on parasites were not recorded in the Ythan estuary web. This means that while analyses of species roles had a sample size of *n* = 35 (seven sites, five types of species roles), analyses of link roles had a sample size of only *n* = 33 (seven sites for most link types, six sites for predation among parasites and target predation on parasites).

### Quantifying differences in the distribution of roles

#### Median roles

We first visualized the median roles of parasites with and without concomitant predation alongside of those of the three free‐living trophic groups. To do this, we performed a correspondence analysis using the function cca from the package vegan (Oksanen *et al*. 2012) in R (R Development Core Team 2014). Using correspondence analysis of species roles also allowed us to examine the axes along which most variation between roles occurred. We used the same procedure to visualize the median roles of different types of links and the axes along which link roles varied.

To compare median roles, we used a nonparametric permutational multivariate analysis of variance (permanova) (Anderson 2001) across the full set of normalized species (or link) roles. Recall that as we have defined them here, roles are multidimensional descriptions; the spatial median of the roles in a given group thus describes the ‘typical’ role for that group. For species, we compared median roles across trophic groups (T, I, B, P and P${}_{c}$). We conducted a similar permanova analysis comparing median roles across link types (F→F, F→P, P→P, P$\overset{t}{\to }$F and P$\overset{c}{\to }$F). All comparisons of median roles were conducted using the adonis function from the vegan package (Oksanen *et al*. 2012) in R (R Development Core Team 2014).

Like the traditional anova, the permanova first calculates the distance between all pairs of observations and then compares among‐group distances to within‐group distances following a pseudo‐*F* statistic (Anderson 2001). Importantly, a permanova does not assume that the data follow any particular distribution. Instead, a *P*‐value for the test statistic is calculated by directly permuting the raw data (Anderson 2001). Since we were most interested in differences between types of species (or links) and not between different communities, we stratified permutations by community. That is, roles were shuffled randomly within a community but the complete set of roles for that community was not changed by the permutation process. In this way, we compared observed distances only to those that could be randomly generated from the same community, controlling for possible effects of changes in species richness or other properties between communities.

The distance metric used in a permanova helps to define the null hypothesis being tested (Anderson 2006). We used Bray–Curtis dissimilarity between roles as our distance metric because it has proven useful for other ecological questions (Legendre & Legendre 2012) and also has specific properties that make it well suited for our purposes. In particular, Bray–Curtis dissimilarity measures differences between the roles based only on positions in which at least one of the species (or links) appears and hence is not affected by ‘double zeros’ in the data (Legendre & Legendre 2012). This means that species (or links) that appear in few positions are not considered more similar to each other due to the large number of shared zero frequencies. In addition, we wished to avoid a situation in which two species involved in different numbers of links would be considered to have different roles even if they occurred with the same frequencies across all motif positions. We therefore calculated dissimilarities based on relative positional frequencies rather than absolute frequencies (i.e. the number of times a species or link appeared in each position divided by the number of times it appeared in any position).

### Role dispersion

In addition to comparing median roles across communities, we explored the dispersion of roles about these median roles using the function betadisper from the package vegan (Oksanen *et al*. 2012) in R (R Development Core Team 2014). As when comparing median roles, we used Bray–Curtis dissimilarity to measure the dispersion of roles within a community around their group median. We were then able to compare the scaling relationships between role dispersion and species or link richness across communities. We hypothesized that role dispersion of a given type of species or link could increase with the number of those species or links observed at an individual community, indicating that each species and link fills a novel structural role. To determine the relationships between the number of species (or links) of a type at a community and the mean dispersion of roles for that species type at that community, we used a linear regression fit using the function lm in R (R Development Core Team 2014).

### Role diversity

We also measured the diversity of unique roles within a community for each group of species or links. To do this, we used a heuristic optimization method to identify clusters of species (or links) that appear in the same motif positions more often than one would expect by chance (Guimerà, Sales‐Pardo & Amaral 2007; Sales‐Pardo *et al*. 2007; Stouffer *et al*. 2012; Appendix S3, Supporting information). Each cluster was interpreted as a unique role phenotype.

As with dispersion, we then compared the scaling relationships between role diversity and species or link richness across communities. We expected diversity to increase with species or link richness, implying that each species or link adds to the niche space of its food web. To quantify this possible relationship between the number of species or links and the number of roles in a community, we used a generalized linear model with a Poisson distribution and logarithm link function fit using the function glm in R (R Development Core Team 2014).

## Results

### Median roles

**Figure 3:**
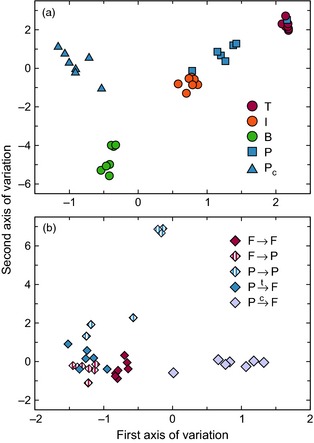
The median roles of species and links vary predictably by type. (a) Within the seven different communities, the different types of species have different median roles, shown here with respect to their location along their first two correspondence analysis axes. The first correspondence analysis axis for species roles described 64·9% of their total variance, and the second axis described 13·0%. When concomitant links are excluded, parasites (P) tend to have roles similar to those of top predators (T). When concomitant links are added, however, parasites' (P${}_{c}$) roles are much more similar to those of basal resources (B). Intermediate species' (I) roles were between those of B and T species. (b) Different types of links also have different median roles, again shown with respect to their first two correspondence analysis axes. The first correspondence analysis axis for links described 60·7% of their total variance, and the second axis described 15·2%. While there is some overlap between roles, concomitant predation links and predation between parasites mainly varied along the first axis while predation between free‐living species, parasitism and target predation on parasites mainly varied along the second axis.

We found that both different trophic groups and different link types have different median roles (see Appendix S4 for more details). Both P${}_{c}$ roles and the roles of P$\overset{c}{\to }$F links were separated from the roles of other types of species or links, respectively, along the first correspondence analysis axis (Fig. 3). This axis corresponded to a division between motifs that include only one‐way interactions and those that include at least one two‐way interaction (Fig. S3), with P${}_{c}$ roles and the roles of P$\overset{c}{\to }$F links being found more often in motifs including at least one two‐way interaction.

### Dispersion & diversity of species roles

**Figure 4:**
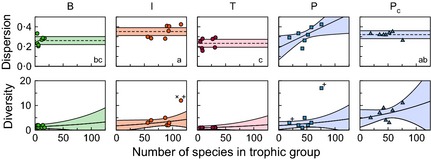
The influence of the number of species in a trophic group on the dispersion and diversity of species roles differed between free‐living species and parasites. (Top row) Role dispersion increased with number of species for parasites without concomitant links (*P* = 0·036). The dispersion of the roles of all other species types did not vary with species richness (dashed lines). The roles of intermediate consumers were most dispersed, followed by those of parasites with concomitant links, basal resources and top predators. Letters in the lower right of each panel indicate groups based on mean dispersions of each type of role (Tukey's HSD test with critical value = 4·11, α = 0·05, and d.f. = 29). Roles with the same letter do not have significantly different mean dispersions. (Bottom row) Role diversity increased with increasing species richness for all types of species (*P* = 0·003), and the estimated rate of increase was the same for all species types. For any given species richness, parasites with concomitant links had more diverse roles than any other type of species, followed by intermediate consumers, parasites without concomitant links, basal resources, and top predators (Tukey's HSD test with critical value = 4·14, α = 0·05, d.f. = 26). In both rows, shaded regions represent 95% confidence regions for the predicted dispersion or diversity after the removal of statistical outliers (indicated by ‘+'s) where applicable. Refer to Appendix S5 for details of the regressions.

Comparing the underlying variation of species roles, we found that dispersion was not affected by species richness for B, I, T, and P${}_{c}$ roles ($t_{28}\;=\;1\cdot 563$, *P* = 0·129; Fig. 4; for details of the regression see Appendix S5). P${}_{c}$ roles were significantly more dispersed than T roles but had similar dispersion to other types of roles (Tukey's HSD test with critical value = 4·11, α = 0·05, and d.f. = 29). Unlike all other types of species roles, dispersion of P roles increased with species richness ($t_{29}\;=\;2\cdot 195$, *P* = 0·036; Appendix S5).

The diversity of distinct roles in a trophic group increased with the number of species in that group, but the strength of this relationship did not vary across groups (Fig. 4). For any given number of species, P${}_{c}$ roles were significantly more diverse than those of other types of species (*z* = 5·632, *P* < 0·001; Appendix S5). P roles were significantly more diverse than T roles but their diversity overlapped with those of I and B roles (Tukey's HSD with critical value 4·14, α = 0·05, and d.f. = 26).

### Dispersion & diversity of link roles

**Figure 5:**
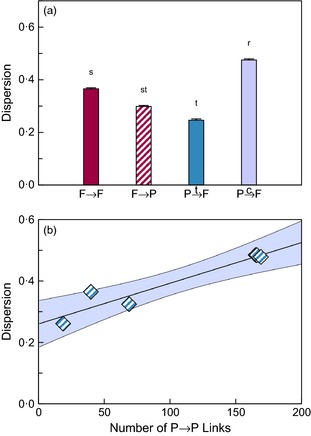
Dispersion of link roles varied across link types while diversity did not. (a) The roles of concomitant predation links (P$\overset{c}{\to }$F) were most dispersed followed by those of predation among free‐living species (F→F), parasitism (F→P) and target predation on parasites (P$\overset{t}{\to }$F). For these link types, the dispersion of link roles was not related to the number of links in a community. (b) Dispersion of the roles of links describing predation between parasites, on the other hand, increased with the number of such links in a community. In (a), the different letters indicate significantly different dispersions and the error bars depict 95% confidence intervals about the mean. Letters above each bar indicate groups based on mean dispersions; types of link with different letters have significantly different dispersions (Tukey's HSD test with critical value 4·13, α = 0·05, d.f. = 27). In (b), the shaded region represents a 95% confidence region for predicted dispersion. Refer to Appendix S5 for details of the regressions.

Dispersion of the roles of P→P links was positively related to the number of those links in a community ($t_{27}\;=\;4\cdot 195$, *P* < 0·001; Fig. 5b; Appendix S6) and was independent of the number of links for all other link types. Of those, the roles of P$\overset{c}{\to }$F links were the most widely dispersed, followed by those of F→F links, F→P links and P$\overset{t}{\to }$F links (Tukey's HSD test with critical value 4·13, α = 0·05, and d.f. = 27; Fig. 5a). In contrast to the diversity of species roles, the diversity of unique link roles did not vary with the number of links of that type in a community (Fig. S6), nor did it differ across types of links (Tukey's HSD test with critical value 4·10, α = 0·05, and d.f. = 28).

## Discussion

Parasites' unique life histories and ways of feeding suggest that they should interact with other species differently than free‐living species (Marcogliese & Cone 1997; Lafferty, Dobson & Kuris 2006; Lafferty *et al*. 2008; Warren *et al*. 2010; Thieltges *et al*. 2013). Despite these important morphological and behavioural differences, a previous study comparing versions of food webs including and excluding parasites found that webs including both types of species but not concomitant predation have similar structural properties to similarly sized webs composed of free‐living species only (Dunne *et al*. 2013a). This indicates that differences between free‐living species and parasites as consumers do not translate to the network level (Dunne *et al*. 2013a). Nevertheless, webs including free‐living species, parasites and concomitant predation links do indeed have different structures from other webs, suggesting that it is parasites' unique positions as concomitant resources that have the greatest effects on network structure, including effects on properties such as connectance which have been linked to robustness (Dunne, Williams & Martinez 2002a; Dunne *et al*. 2013a). In order to examine this inference in greater detail, here we have examined food web structure from the perspective of species and the links between them. We have thus been able to systematically uncover the ways in which free‐living species, parasites, and the multiple types of links between them differ in the broader food web context.

At the species level, our results reaffirmed the impact of links in which parasites are concomitant resources on network structure (Poulin *et al*. 2013; Thieltges *et al*. 2013). The roles of parasites excluding concomitant predation were most similar to those of top predators and intermediate consumers. One potential explanation for the similarity of parasites' roles to those of free‐living intermediate consumers could be the aggregation of parasite life stages. While free‐living intermediate consumers may experience predation during any time of life, parasites have very few consumers except during free‐living life stages. Although a stage‐specific analysis is beyond the scope of the present work, this suggests that the structural roles of different parasite life stages could range from those of free‐living basal resources (for non‐feeding stages with consumers) through to those of free‐living top predators (for parasitic stages that are not affected by other parasites in the same host). Nevertheless, when concomitant predation was included, the roles of parasites were distinct from those of any other type of free‐living species. This suggests that the network‐level effects of concomitant predation may truly be due to changes in the roles of parasites themselves.

In addition to affecting the median roles of parasites, the inclusion of concomitant predation greatly altered the distribution of parasites' roles. Specifically, adding concomitant predation increased role variability in parasite‐poor communities to a similar level to that of parasite‐rich communities, such that parasites' roles appeared saturated when concomitant predation was included and unsaturated when they were not. This apparent homogenizing effect of concomitant predation may arise from the fact that these links bind the roles of parasites to those of their hosts, creating intimate structural similarities. In parasite‐poor communities, it is likely that few parasites share common hosts and therefore common concomitant predation links. As parasites ‘inherit’ role variability from their hosts via concomitant predation, less overlap in host ranges among parasites may lead to greater dispersion of their roles.

Unlike role dispersion which was saturated for most trophic groups, role diversity increased with number of species for all groups. This implies that, while species roles are similarly predictable on the basis of species type regardless of the size of the food web, roles overall do not become more redundant as the number of species in the web increases. This observation fits in well with the suggestion that there is no single way to configure a stable community (May 2001). Contrary to models of stable ecosystems where greater diversity begets greater niche overlap in order to use resources as efficiently as possible, in unstable systems each species' niche may have to be distinct if it is to withstand disturbances (May 2001). Beyond this overall lack of saturation, P${}_{c}$ roles were more diverse than other types of roles for a given number of species in the trophic group. Lower redundancy in P${}_{c}$ roles despite their similar dispersion to other role types could be a result of the different potential outcomes of concomitant predation for the parasite. While concomitant predation is always fatal for the host the parasite may, for certain predators, be able to infect the predator and use it as its next host. For many parasites, such ‘trophic transmission’ is an essential part of the life cycle (Thieltges *et al*. 2013), and it is possible that the roles of such links differ from those of concomitant predation links in which the parasite is destroyed. This lack of redundancy, coupled with the increase in role dispersion resulting from including concomitant predation, means that parasites should have widely varying effects on network structure. This in turn implies that parasites can generate a variety of effects on population dynamics and energy flows through their communities. In particular, lack of redundancy means that any effects of fluctuations in the population of one parasite (e.g. on host mortality) are unlikely to be compensated for by another parasite with a similar role.

To further clarify the impact of different types of links, we considered the roles of links directly. The dispersion of link roles generally appeared to be saturated – that is, independent of the number of a given type of links present in a network. This suggests that there were sufficient links in each community to occupy the entire role space for most types of links. Given the saturation of role dispersion for most types of species, this is not surprising. The only type of link for which role dispersion was not saturated was predation among parasites. This type of link includes hyperparasitism, predation among free‐living stages of parasites and attack by one parasite on others within the same host, with or without consumption (Hechinger *et al*. 2011b). This variety of types of feeding and interaction locations might explain the apparent tendency for links describing predation among parasites to be increasingly distinct from the group median. Surprisingly, this variability in link roles does not appear to be linked to a greater diversity of unique role phenotypes.

Dispersion, conversely, differed among link types with the roles of concomitant predation links being the most variable. While concomitant predation ties the roles of parasites to those of their hosts, the roles of these links are non‐trivially tied to the roles of the predation links that lead to them. Alternatively, it is possible that the wide variety of outcomes of concomitant predation for both parasite and consumer (Thieltges *et al*. 2013) leads to these links having inherently more variable roles. Were that the case, however, we could expect a greater diversity of unique roles for these links as well as greater diversity, which we did not observe. It therefore appears that, by combining predation with parasitism, concomitant predation is simply less predictable than other types of interactions. This may mean that the consequences of concomitant predation for energy flows or population dynamics are similarly unpredictable.

## Conclusions

Our species‐centric and link‐centric perspectives allow us to robustly identify how and where the contributions of parasites to network structure differ from those of different types of free‐living species. Within a complex food web, it is common to characterize species' structural roles in terms of the organization of their direct and indirect links with other species (Luczkovich *et al*. 2003; Olesen *et al*. 2007; Allesina & Pascual 2009). As we show here, the structural roles of links can also be characterized by the pair of species that make them up and, by extension, all other links those species participate in. Though both perspectives build from the same fundamental information, our analysis demonstrates that they are not equivalent and instead provide a complementary picture of the building blocks of food web structure.

Overall, our results reinforce the idea that concomitant predation plays a disproportionately important part in determining the structure of food webs (Dunne *et al*. 2013a; Poulin *et al*. 2013) and that it places considerable constraints on the median roles of parasites while simultaneously increasing the variability about these median roles. This implies that concomitant predation not only affects the ways in which parasites in general affect community functions and stability but that it decreases the redundancy of each species' contribution to those effects. Historically, concomitant predation has often been omitted from food webs, either because it is assumed to be energetically insignificant (Thieltges *et al*. 2013) or because it is inherently difficult to directly observe (Marcogliese & Cone 1997). The structural implications of these links as shown here, as well as their prevalence within food webs (Thieltges *et al*. 2013), potential energetic implications (Lafferty, Dobson & Kuris 2006; Hechinger *et al*. 2011a; Thompson *et al*. 2013) and importance as sources of either mortality or trophic transmission (Lafferty, Dobson & Kuris 2006; Thieltges *et al*. 2013) for parasites mean that they should no longer be ignored. Finally, as concomitant predation links reveal the deep intimacy between hosts and parasites, they provide a critical lens through which to examine the many ways in which parasite–host and predator–prey interactions are linked.

## Data accessibility

Food webs used in this study are available from the Dryad Digital Repository: http://dx.doi.org/10.5061/dryad.b8r5c (Dunne *et al*. 2013b).

## Supporting information


**Appendix S1.** References and description of food webs.
**Appendix S2.** Methods for quantifying species and link roles.
**Appendix S3.** Methods for quantifying role dispersion and diversity.
**Appendix S4** Detailed results for median roles.
**Appendix S5** Model selection for analysis of dispersion and diversity of species roles.
**Appendix S6** Model selection for analysis of dispersion and diversity of link roles. Figure showing link role diversity.

**Fig. S1.** Three‐species motifs with unique positions numbered.

**Fig. S2.** Three‐species motifs with unique links numbered.

**Fig. S3.** Alignment of unique positions and unique links with major axes of variation in median roles.

**Fig. S4.** Diversity of unique link roles did not vary across link types or with number of links in a community.
