# Supplementary material for: Concomitant predation on parasites is highly variable but constrains the ways in which parasites contribute to food web structure
Source: J Anim Ecol. 2015 Jan 8;84(3):734–44. doi: 10.1111/1365-2656.12323 (PMC4964941; doi:10.1111/1365-2656.12323)
Supplement: Supplementary file 1 — Appendix S1. References and description of food webs. Appendix S2. Methods for quantifying species and link roles. Appendix S3. Methods for quantifying role dispersion and diversity. Appendix S4 Detailed results for median roles. Appendix S5 Model selection for analysis of dispersion and diversity of species roles. Appendix S6 Model selection for analysis of dispersion and diversity of link roles. Figure showing link role diversity. [file JANE-84-734-s001.pdf]

Supporting Information

Alyssa R. Cirtwill *[a] and Daniel B. Stouffer †[a]

[a]School of Biological Sciences, University of Canterbury,
Christchurch, New Zealand

†Corresponding author: daniel.stouffer@canterbury.ac.nz

*alyssa.cirtwill@canterbury.ac.nz
†daniel.stouffer@canterbury.ac.nz

Materials & Methods

S1. Additional References and Description of Food Webs

Table S1: Locations and original sources for food-web datasets. The Ythan web used is version 3 from Huxham, Beaney & Raffaelli (1996). Following Huxham, Beaney & Raffaelli (1996), species 100 in this web was removed as it is an animal with no recorded resources in the food web. This also resulted in the removal of one link 100 → 85 where species 100 appeared as a resource.

Site	Source	Location
Bahia	Hechinger *et al.* (2011)	Bahia Falsa, Baja California<br>Mexico
Carpinteria	Hechinger *et al.* (2011)	Carpinteria Salt Marsh, California<br>USA
Estero	Hechinger *et al.* (2011)	Estero de Punta Banda, Baja California<br>Mexico
Fjord	Zander *et al.* (2011)	Flensburg Fjord, Baltic Sea<br>Germany/Denmark
Otago	Mouritsen *et al.* (2011)	Otago Harbour<br>New Zealand
Sylt	Thieltges *et al.* (2011)	Sylt Tidal Basin, North Sea<br>Germany/Denmark
Ythan	Huxham, Beaney & Raffaelli (1996)	Ythan Estuary, Scotland<br>UK

Trophic groups of free-living species were defined based on the free-living webs. Top predators (T) were defined as species with prey but no predators, basal resources (B) as species with predators but no prey, and intermediate consumers (I) were all remaining species (that is species with both predators and prey). Cannibalistic species were considered to be intermediate consumers, as some individuals serve as prey to their conspecifics even if they are not prey to other species (Williams & Martinez, 2000). Parasites were defined by the authors of the original

food webs, and included species ranging from apicomplexan and ciliate protozoans to nematode, trematode, and cestode worms to parasitic copepods (Dunne *et al.*, 2013; Huxham, Beaney & Raffaelli, 1996; Hechinger *et al.*, 2011; Mouritsen *et al.*, 2011; Thieltges *et al.*, 2011; Zander *et al.*, 2011). Any species with both parasitic and free-living life stages was considered a parasite.

Table S2: Representation of each type of species across the different food webs. Type "free-living" refers to webs with free-living species only while type "par & con" refers to "parasite" and "concomitant" webs which include parasites and free-living species. S refers to the total species richness in each web. $\%_F$, $\%_T$, $\%_I$, $\%_B$, and $\%_P$ refer to the proportion of species that are free-living, top predators, intermediate consumers, basal resources, and parasites, respectively.

Site	Type	S	$\%_F$	$\%_T$	$\%_I$	$\%_B$	$\%_P$
Bahia	free-living	119	100	7	79	14	0
Bahia	par & con	171	70	5	55	10	30
Carpinteria	free-living	107	100	5	84	11	0
Carpinteria	par & con	165	65	3	55	7	35
Estero	free-living	138	100	7	83	10	0
Estero	par & con	214	64	4	54	6	36
Flensburg	free-living	77	100	12	80	8	0
Flensburg	par & con	123	62	7	50	5	38
Otago	free-living	123	100	26	71	3	0
Otago	par & con	142	87	23	61	3	13
Sylt	free-living	126	100	21	74	5	0
Sylt	par & con	161	78	17	58	3	22
Ythan	free-living	91	100	34	62	4	0
Ythan	par & con	133	68	23	42	3	32

Table S3: Frequency of different types of links across the different food webs. L refers to the total number of links in each web while $F \to F$, $P \to F$, $P \to P$, $F \xrightarrow{t} P$, and $F \xrightarrow{c} P$ to the number of links describing predation among free-living species, parasitism, predation between parasites, target predation of free-living species on parasites, and concomitant predation on parasites, respectively. Note that neither $F \xrightarrow{t} P$ nor $P \to P$ links were observed in the Ythan web.

Site	Type	L	$F \to F$	$F \to P$	$P \to P$	$P \xrightarrow{t} F$	$P \xrightarrow{c} F$
Bahia	free-living	1075	1075	0	0	0	0
Bahia	parasite	2232	1075	807	165	185	0
Bahia	concomitant	3765	1075	807	165	185	1533
Carpinteria	free-living	963	963	0	0	0	0
Carpinteria	parasite	2180	963	755	166	296	0
Carpinteria	concomitant	3762	963	755	166	296	1582
Estero	free-living	1647	1647	0	0	0	0
Estero	parasite	3324	1647	835	169	673	0
Estero	concomitant	5805	1647	835	169	673	2481
Fjord	free-living	577	577	0	0	0	0
Fjord	parasite	966	577	271	40	78	0
Fjord	concomitant	1428	577	271	40	78	462
Otago	free-living	1200	1200	0	0	0	0
Otago	parasite	1481	1200	173	19	89	0
Otago	concomitant	1852	1200	173	19	89	371
Sylt	free-living	1047	1047	0	0	0	0
Sylt	parasite	1944	1047	552	69	276	0
Sylt	concomitant	3033	1047	552	69	276	1089
Ythan	free-living	416	416	0	0	0	0
Ythan	parasite	593	416	177	0	0	0
Ythan	concomitant	1268	416	177	0	0	675

S2. Quantifying Species' and Links' Roles

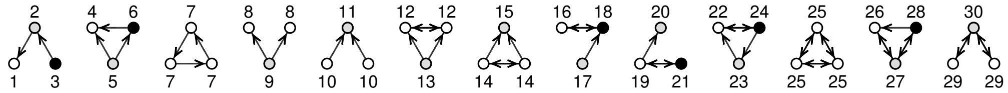

Figure S1: Three-species motifs with unique positions numbered.

Interactions between species are a direct consequence of the motif structure of a food web. Motifs are the set of 13 three-species subwebs describing all possible interaction patterns of three species (Milo *et al.*, 2002; Stouffer *et al.*, 2007, Fig. S1). Each motif contains one or more unique positions, indicating a unique way in which a species' interactions are organised in that motif (e.g., the top predator, intermediate consumer, and resource in a three- species food chain) (Stouffer *et al.*, 2012). In the 13 three-species motifs, there are 30 such positions (Kashtan *et al.*, 2004; Stouffer *et al.*, 2012). Similarly, there are 24 unique link types connecting species (Fig. S2). By counting the frequency c_{ij}^{w} with which each species i in community s in web type w (i.e., free-living, parasite, or concomitant) occurs in each position j, we obtained a vector $\overrightarrow{f_{si}^{w}}$ describing the overall role of that species within its food web,

$$\overrightarrow{f_{si}^{w}} = \{c_{i1}, c_{i2}, ..., c_{i29}, c_{i30}\}_{s}^{w} . \tag{1}$$

The same process was used to determine the roles of links between species,

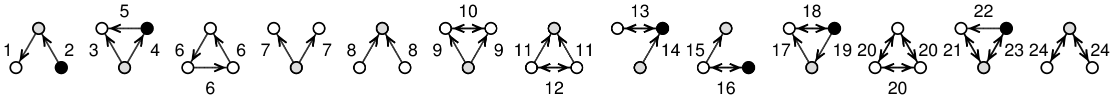

Figure S2: Three-species motifs with unique links numbered.

giving a vector

$$\overrightarrow{f_{sl}^{w}} = \{c_{l1}, c_{l2}, ..., c_{l23}, c_{l24}\}_s^w . \qquad (2)$$

that describes the role $\overrightarrow{f_{sl}^{w}}$ for each link l in community s in web type w.

S3. Role Dispersion & Diversity

As described in the main text, we quantified the distribution of species' and links' roles by their role dispersion and role diversity (Fig. 2, main text). In order to quantify role diversity, we first needed to identify subsets of species (or links) that have statistically-similar motif-based roles; that is, clusters of species (or links) that appear in the same motif positions more often than one would expect by chance. To perform a clustering of this nature, we followed a recently-proposed method that is an extension of community detection algorithms for complex networks to the case of detecting groups of nodes in bipartite networks with weighted edges (Sales-Pardo *et al.*, 2007; Stouffer *et al.*, 2012). Here, the bipartite network consists of each species (or link) in our dataset on one side and the different motif positions on the other. Each edge in this network is weighted by the frequency c_{sij}^{w} with which the species or link i in community s in web type w occupies position j. The

clustering algorithm consists of maximising an objective function M (referred to as “modularity”) that is high when nodes in the same cluster tend to occupy the same positions with similar frequencies and low otherwise (Stouffer *et al.*, 2012).

We used a stochastic and heuristic optimisation method known as simulated annealing (Kirkpatrick, Gelatt & Vecchi, 1983) to cluster nodes (species or links) while maximising modularity (Sales-Pardo *et al.*, 2007; Girvan & Newman, 2002). Since this procedure is not always guaranteed to find a global optimum, and since we are most interested in the expected variety of clusters per group as a proxy for role diversity, we performed this modularity maximisation 100 separate times for roles of species and links in each community. As with dispersion, we included the roles of free-living species from the “free-living” web as well as the roles of parasites from both the “parasite” and “concomitant” webs. We then calculated the weighted average number of clusters containing each type of species (or link) across the 100 modularity-maximised clusterings following

$$\hat{N}_j = \sum_i p_i N_{ij} \quad , \quad p_i = e^{M_i} / \sum_k e^{M_k} \, , \tag{3}$$

where M_i is the modularity of a given clustering i, $\sum_k e^{M_k}$ is the sum of modularities over all k clusterings, and p_i is the relative probability of obtaining a clustering i weighted by its modularity; N_{ij} is the number of clusters containing

species type j in clustering i, and $\hat{N}_j$ is the weighted average of the number of clusters containing species (or link) type j (Sales-Pardo *et al.*, 2007). We assume that each cluster represents a unique structural role, therefore this average number of clusters provides an estimate of the role diversity for each type of species and links.

Results

S4. Median Roles

When comparing across different types of species, we found that trophic group was a significant predictor of median roles, as hypothesised ($F_{4,1432} = 218.15$, p= 0.001; Fig. 3A, main text). The P roles were between those of I and T free-living species, and they slightly overlapped with each. The P_c roles, in contrast, were distinct from all other role types. They were separated from T, I, and P roles along the first correspondence analysis axis (which accounted for 64.9% of total variance in species roles) and separated from B roles along the second correspondence analysis axis (which explained 13.0% of total variance).

The first axis corresponds mainly to a split between positions in motifs containing only one-way interactions and positions in motifs with at least one two-way interaction (Fig. S3A). T, I, and P roles are associated with a greater frequency of one-way motifs, while P_c roles are associated with a greater frequency of two-way

motifs. The second axis was largely defined by the frequencies of positions 3 and 9 (Fig. S2). Position 3 represents the base of a three-species food chain, while position 9 represents a species which is preyed upon by two other species (apparent competitors). These positions are more frequent in B roles and less frequent in other types of roles.

When comparing different types of links, we found that link type significantly predicted median roles ($F_{4,20908} = 1018.75$, $p < 0.001$; Fig. 3B, main text). There was a great deal of overlap between the median roles of F→P and F→F links while the median roles of P→P links were highly variable across communities. In general, the roles of P$\xrightarrow{\text{c}}$F and P→P links showed more variation along the first principal-component axis (which accounted for 60.7% of total variance in link roles) while the roles of F→F links, F→P links, and P$\xrightarrow{\text{t}}$F links showed more variation along the second principal-component axis (which accounted for 15.2% of total variance).

As with species roles, the first correspondence axis corresponds to a split between one-way interactions and two-way interactions (Fig. S3B). Two-way interaction positions were more frequent in the roles of concomitant predation links and less frequent in other groups. The second axis corresponds to mainly to link positions 10 and 12, which represent species with a common prey that consume each other and species which consume each other and have a common predator,

respectively (Fig. S3). These link positions are most common in links describing predation among parasites.

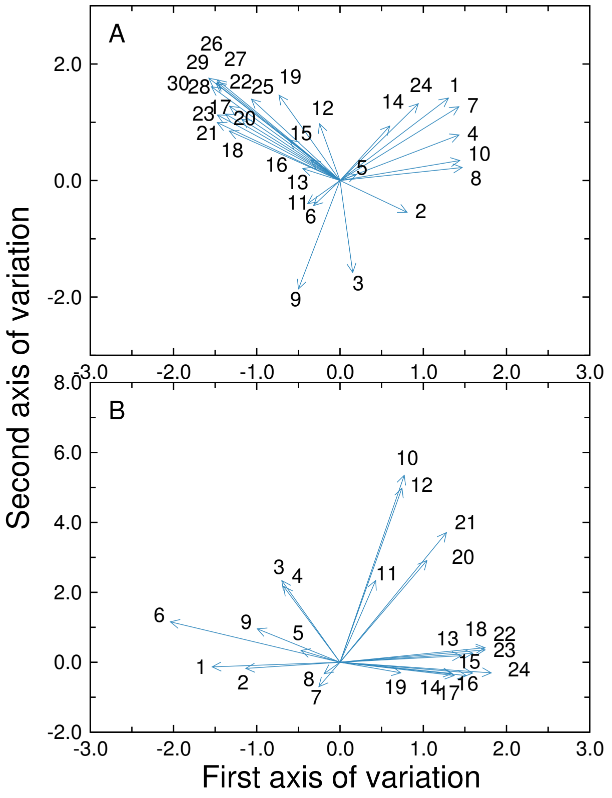

Figure S3: The major axes of variation for median roles demonstrated key differences in the roles of different types of species and links. **(A)** The first major axis of variation for species roles corresponded to a split between positions in motifs containing only one-way interactions and positions in motifs containing at least one two-way interaction. This axis separates the roles of parasites including concomitant predation from other types of roles (Fig. 3A). The second major axis was largely defined by positions representing the base of a three-species food chain (3) and a species with two predators which do not eat each other. These positions are most common in the roles of basal resources. **(B)** The first major axis of variation for link roles also corresponds to a split between positions in motifs that contain only one-way interactions and those in motifs containing at least one two-way interaction. Positions associated with two-way interactions were more frequent in the roles of concomitant predation links than in other role types (Fig. 3B). The second axis is largely determined by two positions representing mutual predation between species with a common prey or common predator. These positions are most common in the roles of links describing predation between parasites.

S5. Species roles

Dispersion

We determined the overall relationship between species-richness and role dispersion using the model

$$\sigma_{gs} = \beta_1 B_g + \beta_2 I + g + \beta_3 T_g + \beta_4 P_g + \beta_5 P_{\mathrm{cg}} + \beta_6 N_{gs} + \beta_6 P_g N_{gs}\,. \tag{4}$$

where σ_{gs} is the dispersion of group g (B, I, T, P, or $\mathrm{P_c}$) in community s (e.g., Ythan), B_g, I_g, T_g, P_g, and P_{cg} are dummy variables that equal 1 if g is the corresponding group type (i.e., B_g=1 if g represents the roles of basal resources), N_{gs} is the number of species N in group g at community s, and $P_g N_{gs}$ represents the number of species N in group g at community s if g represents the roles of parasites without concomitant predation links.

We then removed the non-significant overall effect of species richness (Table S4), leaving the model,

$$\sigma_{gs} = \beta_1 B_g + \beta_2 I_g + \beta_3 T_g + \beta_4 P_g + \beta_5 P_{\mathrm{cg}} + \beta_6 P_g N_{gs}\,, \tag{5}$$

which was used to compare the dispersions of B, I, T, and $\mathrm{P_c}$ roles as well as the slope of P role dispersion over species richness.

Table S4: Standardised effects, t-values, and p-values for all terms included in models 1 and 2, as well as the F-statistic, degrees of freedom, and p-value of each model overall

	Model 1			Model 2		
Parameter	Effect	t-value	p-value	Effect	t-value	p-value
B	0.251	11.903	<0.001	0.261	12.703	<0.001
I	0.255	3.961	<0.001	0.352	17.133	<0.001
T	0.213	9.050	<0.001	0.233	11.344	<0.001
P	0.189	3.157	0.004	0.189	3.081	0.005
P_{c}	0.268	6.825	<0.001	0.320	15.611	<0.001
N_{gs}	0.001	1.563	0.129		NA	
$P_g N_{gs}$	0.002	1.128	0.269	0.003	2.195	0.036
F-statistic		160.6			178.1	
Degrees of freedom		7, 28			6, 29	
Overall p-value		<2.2e-16			<2.2e-16	

Diversity

We tested the effect of species richness on role diversity using the model,

$$\delta_{gs} = \beta_1 B_g + \beta_2 I_g + \beta_3 T_g + \beta_4 P_g + \beta_5 P_{\mathrm{cg}} + \beta_6 N_{gs}\,, \quad (6)$$

where δ_{gs} is the role diversity of trophic group g in community s and all other symbols are as in the dispersion models above. Only $\mathrm{P_c}$ roles had a diversity significantly different from zero and there was no significant effect of species richness. This model was also used in the Tukey's HSD test of mean diversities across groups, as the reduced model used to establish the mean diversity of $\mathrm{P_c}$ roles,

$$\delta_{gs} = \beta_0 + \beta_1 P_{\mathrm{cg}} + \beta_2 N_{gs}\,, \tag{7}$$

did not include intercepts for other role types (Table S5).

S6. Link roles

Dispersion

We examined the effect of link richness on the dispersion of link roles using the model,

$$\sigma_{ls} = \beta_1 F \rightarrow F_l + \beta_2 F \rightarrow P_l + \beta_3 P \xrightarrow{t} F_l + \beta_4 P \xrightarrow{c} F + \beta_5 P \rightarrow P_l + \beta_6 N_{ls} + \beta_7 P \rightarrow P_l N_{ls}\,, \tag{8}$$

Table S5: Standardised effects, z-values, and p-values for all terms included in models 3 and 4, as well as the AIC and degrees of freedom of each model overall

	Model 3			Model 4		
Parameter	Effect	t-value	p-value	Effect	t-value	p-value
Intercept		NA		0.189	0.802	0.422
B	0.291	0.901	0.368		NA	
I	0.566	0.892	0.372		NA	
T	-0.128	-0.320	0.749		NA	
P	0.617	1.407	0.159		NA	
P_{c}	1.558	4.081	<0.001	1.151	5.632	<0.001
N	0.007	1.000	0.317	0.012	2.968	0.003
AIC		122.56			108.83	
Degrees of freedom		26			29	

where σ_{ls} is the dispersion of the roles of link type l in community s, $F \rightarrow F_l$, $F \rightarrow P_l$, $P \xrightarrow{t} F_l$, $P \xrightarrow{c} F_l$, and $P \rightarrow P_l$ are dummy variables that are equal to 1 if link type l is the relevant type (i.e., $F \rightarrow F_l$=1 for $F \rightarrow F$ links) and 0 otherwise, N_{ls} is the number of links of type l in community s, and $P \rightarrow P_l N_{ls}$ is an additional effect of link richness specific to $P \rightarrow P$ roles, only the model above which includes the interaction between link richness and $P \rightarrow P$ roles showed any significant effect of link richness on link role dispersion. This model was used to conclude that link richness does not affect the dispersion of $F \rightarrow F_l$, $F \rightarrow P_l$, $P \xrightarrow{t} F_l$, and $P \xrightarrow{c} F_l$ roles.

We then used the reduced model,

$$\sigma_{ls} = \beta_1 F \rightarrow F_l + \beta_2 F \rightarrow P_l + \beta_3 P \xrightarrow{t} F_l + \beta_4 P \xrightarrow{c} F_l + \beta_5 P \rightarrow P_l + \beta_7 P \rightarrow P_l N_{ls}\,, \quad (9)$$

which includes an effect of link richness for $P \rightarrow P$ roles only, to calculate the confidence intervals in Fig. 6 (main text). The best parameter estimates returned by the two models were very similar (Table S6).

Diversity

Finally, we determined that there was no effect of link richness on link role diversity using the model

$$N_{ls} = \beta_1 F \rightarrow F_l + \beta_2 F \rightarrow P_l + \beta_3 P \xrightarrow{t} F_l + \beta_4 P \xrightarrow{c} F_l + \beta_5 P \rightarrow P_l + \beta_7 N_{ls}\,, \quad (10)$$

where N_{ls} is the role diversity for link type l in community s and all other symbols are as above. We then used the model

$$\delta_{ls} = \beta_1 F \rightarrow F_l + \beta_2 F \rightarrow P_l + \beta_3 P \xrightarrow{t} F_l + \beta_4 P \xrightarrow{c} F_l + \beta_5 P \rightarrow P_l\,, \quad (11)$$

to generate confidence intervals in Fig. S4. Although the estimated diversities for each link type differed between models (Table S7), the standard errors on these estimates were large, such that different types of links did not have significantly

Table S6: Standardised effects, t-values, and p-values for all terms included in models 5 and 6, as well as the F-statistic, degrees of freedom, and p-value of each model overall

	Model 5			Model 6		
Parameter	Effect	t-value	p-value	Effect	t-value	p-value
$F \rightarrow F_l$	0.345	12.066	<0.001	0.359	19.342	<0.001
$F \rightarrow P_l$	0.295	13.524	<0.001	0.302	16.282	<0.001
$P \xrightarrow{t} F_l$	0.264	12.504	<0.001	0.267	13.338	<0.001
$P \xrightarrow{c} F_l$	0.450	14.179	<0.001	0.466	25.095	<0.001
$P \rightarrow P_l$	0.262	6.709	<0.001	0.262	6.783	<0.001
N_{ls}	<0.001	0.640	0.528		NA	
$P \rightarrow P_l N_{ls}$	0.001	4.095	<0.001	0.001	4.195	<0.001
F-statistic		260.3			310.4	
Degrees of freedom		7, 26			6, 27	
Overall p-value		<2.2e-16			<2.2e-16	

different role diversities.

Table S7: Standardised effects, z-values, and p-values for all terms included in models 7 and 8, as well as the AIC and degrees of freedom of each model overall

	Model 7			Model 8		
Parameter	Effect	t-value	p-value	Effect	t-value	p-value
$F \rightarrow F_l$	12.147	2.676	0.013	8.616	2.886	0.007
$F \rightarrow P_l$	9.022	3.465	0.015	7.201	2.412	0.023
$P \xrightarrow{t} F_l$	9.483	3.350	0.009	8.533	2.646	0.013
$P \xrightarrow{c} F_l$	14.730	2.929	0.007	10.553	3.535	0.001
$P \rightarrow P_l$	7.507	2.316	0.028	7.133	2.212	0.035
N_{ls}	-0.004	-1.032	0.311		NA	
AIC		237.35			236.63	
Degrees of freedom		27			28	

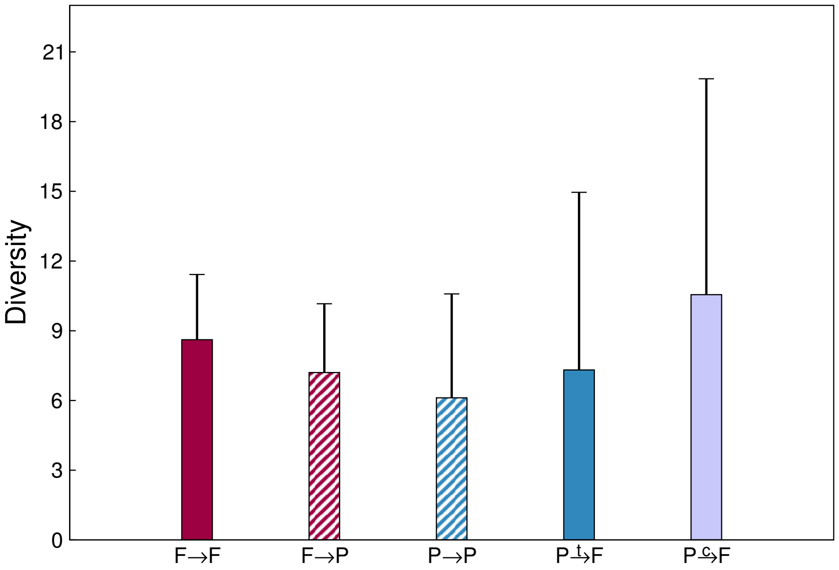

Figure S4: Diversity of unique roles was not related to the number of links in a community for any link type. Diversity of unique roles did not differ across link types.

References

Dunne, J.A., Lafferty, K.D., Dobson, A.P., Hechinger, R.F., Kuris, A.M., Martinez, N.D., McLaughlin, J.P., Mouritsen, K.N., Poulin, R., Reise, K., Stouffer, D.B., Thieltges, D.W., Williams, R.J. & Zander, C.D. (2013) Parasites affect food web structure primarily through increased diversity and complexity. *PLoS Biology*, **11**, e1001579.

Girvan, M. & Newman, M.E.J. (2002) Community structure in social and biological networks. *Proceedings of the National Academy of Sciences of the United States of America*, **99**, 7821–7826.

Hechinger, R.F., Lafferty, K.D., Mclaughlin, J.P., Fredensborg, B.L., Huspeni, T.C., Lorda, J., Sandhu, P.K., Shaw, J.C., Torchin, M.E., Whitney, K.L. & Kuris, A.M. (2011) Food webs including parasites, biomass, body sizes, and life stages for three California/Baja California estuaries. *Ecology*, **92**, 791.

Huxham, M., Beaney, S. & Raffaelli, D. (1996) Do parasites reduce the chances of triangulation in a real food web? *Oikos*, **76**, 284–300.

Kashtan, N., Itzkovitz, S., Milo, R. & Alon, U. (2004) Topological generalizations of network motifs. *Physical Review E*, **70**, 031909.

Kirkpatrick, S., Gelatt, C. & Vecchi, M. (1983) Optimization by simulated annealing. *Science*, **220**, 671–680.

Milo, R., Shen-Orr, S., Itzkovitz, S., Kashtan, N., Chklovskii, D. & Alon, U. (2002) Network motifs: simple building blocks of complex networks. *Science*, **298**, 824–7.

Mouritsen, K.N., Poulin, R., McLaughlin, J.P. & Thieltges, D.W. (2011) Food web including metazoan parasites for an intertidal ecosystem in New Zealand. *Ecology*, **92**, 2006.

Sales-Pardo, M., Guimerà, R., Moreira, A.A. & Amaral, L.A.N. (2007) Extracting the hierarchical organization of complex systems. *Proceedings of the National Academy of Sciences of the United States of America*, **104**, 15224–15229.

Stouffer, D.B., Camacho, J., Jiang, W. & Amaral, L.A.N. (2007) Evidence for the existence of a robust pattern of prey selection in food webs. *Proceedings of the Royal Society of London B*, **274**, 1931–1940.

Stouffer, D.B., Sales-Pardo, M., Sirer, M.I. & Bascompte, J. (2012) Evolutionary conservation of species' roles in food webs. *Science*, **335**, 1489–1492.

Thieltges, D.W., Reise, K., Mouritsen, K.N., Mclaughlin, J.P. & Poulin, R. (2011) Food web including metazoan parasites for a tidal basin in Germany and Denmark. *Ecology*, **92**, 2005.

Williams, R.J. & Martinez, N.D. (2000) Simple rules yield complex food webs. *Nature*, **404**, 180–183.

Zander, C.D., Josten, N., Detloff, K.C., Poulin, R., Mclaughlin, J.P. & Thieltges, D.W. (2011) Food web including metazoan parasites for a brackish shallow water ecosystem in Germany and Denmark. *Ecology*, **92**, 2007.
